# TRIM11, a new target of p53, facilitates the migration and invasion of nasopharyngeal carcinoma cells

**DOI:** 10.1007/s11033-022-07833-z

**Published:** 2022-11-14

**Authors:** Ziyi Zhao, Jinkuang Deng, Ming Lu, Jun Yang, Linlin Chen, DianYuan Li, Yi Sang

**Affiliations:** 1 Jiangxi Key Laboratory of Cancer Metastasis and Precision Treatment, The First Hospital of Nanchang, Nanchang, 330008 China; 2 Jiangxi Engineering Laboratory for the Development and Utilisation of Agricultural Microbial Resources, College of Bioscience and Biotechnology, Jiangxi Agricultural University, Nanchang, 330045 China; 3 Department of Otolaryngology Head and Neck Surgery, The Eight Affiliated Hospital, Sun Yat-Sen University, Shenzhen, 518033 China; 4 The Key Laboratory of Oral Biomedicine in Jiangxi Province, Department of Oral and Maxillofacial Surgery, The Affiliated Stomatological Hospital of Nanchang University, Nanchang, 330006 China; 5 Department of Cardiovascular Surgery, The Affiliated Suzhou Hospital, Nanjing Medical University, Suzhou, 215002 China

**Keywords:** TRIM11, p53, Migration, Invasion, Nasopharyngeal carcinoma

## Abstract

**Background:**

Although tripartite motif-containing protein 11 (TRIM11) is known to be associated with a variety of cancers, its role in nasopharyngeal carcinoma (NPC) is unclear.

**Methods and results:**

To investigate the role of TRIM11 in NPC, TRIM11 was stably overexpressed in 6–10B and CNE2 cells with lentiviral vectors and knocked down in S18 and 5–8F cells using the CRISPR/Cas9 system. Transwell assays and wound-healing assays revealed that TRIM11 facilitated the migration and invasion of NPC cells. Mechanistically, we found that p53 inhibits TRIM11 expression by binding to its promoter.

**Conclusions:**

TRIM11 may serve as a potential diagnostic marker for NPC and has a certain therapeutic value.

## Introduction

Metastasis plays a key role in tumour progression and seriously threatens the lives of cancer patients. Invasion occurs in the early stages of tumour metastasis [1]. NPC, prevalent in Asia, exhibits unique biological behaviours, such as sensitivity to radiotherapy and susceptibility to distant metastasis [2]. Studies have shown that changes in gene expression promote cell migration and invasion. For example, limb-bud and heart (LBH) inhibit the migration and invasion of NPC cells [3]. Hence, elucidating the molecular mechanism of underlying NPC metastasis helps prevent and treat NPC metastasis.

The TRIM (tripartite motif-containing) protein family has been identified as a novel family of Ub E3 ligases, that have three protein domains, namely, the B-Box, RING and coil (RBCC) domains [4]. Many members of the TRIM family influence the biological behaviours of cancer cells. For example, TRIM39 deficiency inhibits tumour progression and autophagic flux in colorectal cancer cells [5], and TRIM37 orchestrates renal cell carcinoma progression [6].

p53 is a transcription factor, recognised as one of the most potent tumour suppressor proteins [7]. In addition to its transcriptional functions, p53 exerts transcription-independent effects in the regulation of apoptosis, necrosis, autophagy, metabolism, and DNA replication and repair [8]. p53 mutation is not common in NPC [9, 10]. Overexpression of p53 by adenoviral vectors has been reported to have anti-NPC effects [11, 12]. Therefore, further revealing the signal transduction processes associated with p53 is very important for the treatment of NPC.

Our results demonstrated that overexpression of TRIM11 enhanced the migration and invasion abilities of NPC cells. Mechanistically, we provided detailed evidence that p53 can bind to the TRIM11 promoter to inhibit its expression.

## Materials and methods

### Cell culture

Human NPC cell lines (5-8F, 6-10B, S18 and CNE2 cells) were obtained from Tiebang Kang (Sun Yat‑Sen University Cancer Center, Guangzhou, China). The 5-8F, 6-10B, S18, and CNE2 cell lines were maintained in DMEM with 5% CO_2_ atmosphere and at 37 °C. The medium was supplemented with 10% fetal bovine serum (FBS). FBS (#10270-106) was from Gibco.

### Plasmids

Human TRIM11 cDNA was constructed into a pSin-puro vector to generate a stable cell line. An S–Flag–SBP (SFB) tag was ligated to the TRIM11 N-terminus to generate the human TRIM11 cDNA construct in the plenti-puro vector (Addgene, #39481). An HA tag was inserted into the N-terminal pcDNA3.1 (+) vector to establish the human p53 cDNA construct.

### Antibodies

An anti-TRIM11 antibody was derived from Sigma-Aldrich (HPA028541). An anti-p53 antibody (DO-7; cat. no. SC-47698) was purchased from Santa Cruz Biotechnology (Dallas, TX, USA).

### Stable lines

In brief, 293T cells were cotransfected with recombinant lentiviruses carrying psPAX2 and pMD2.G plasmids validated by DNA sequencing using Lipofectamine 2000. Cell supernatants containing lentiviral particles were then harvested and added to NPC cell.

### Generation of knockdown cell lines

The lentiviral CRISPR/Cas9-mediated TRIM11 and p53 gene editing vectors were generated by annealing gRNA oligonucleotides and subcloning them into pLentiCRISPRv2 as previously described [13]. Then, 293T cells were cotransfected with recombinant lentiviruses plasmids validated by DNA sequencing carrying the pSPAX2 and pMD2.G plasmids validated by DNA sequencing. Cell supernatants containing lentiviral particles were then harvested and added to NPC cells. KD was verified by Western blotting. The sgRNA sequences were as follows: TRIM11: 5′-TGCGTTGCTGTTCCAAGCCC-3′; p53: sg1: 5′-CACCGTGACTGCTTGTAGATGGCCA-3′; sg2: 5′-CACCGGTGCTGTGACTGCTTGTAGA-3′; sg3: 5′-CACCGGCAGTCACAGCACATGACGG-3′.

### Western blotting

In brief, total protein was extracted from NPC cell lines using RIPA buffer (Focus Bioscience, Shanghai). Proteins of different molecular weights were separated by SDS-PAGE and transferred to polyvinylidene fluoride membranes. Western blotting was performed using ECL Western blotting substrate (Focus Bioscience, Shanghai).

### Luciferase assay

293T cells were transfected with luciferase reporter plasmids in a 12-well plate. A Renilla luciferase reporter plasmid was transfected to normalise the transfection efficiency. After transfection for 24 h, the luciferase activity was detected with a Luciferase Dual Assay Kit (promega, USA). Three independent experiments were conducted.

### Transwell assay

In the migration assay, 3.0 × 10^4^ cells (CNE2 and 6-10B cells) or 1.0 × 10^4^ cells (S18 and 5–8F cells) were added to 300 μl serum-free DMEM. An 8-μm microporous filter (BD Labware) not coated with extracellular matrix was used in these experiments. Then, 700-μl medium with 10% FBS was added to the bottom chamber. After culturing in a 5% CO_2_ atmosphere at 37 °C for 24 h, the cells were stained with 0.1% crystal violet for 40 min, and counted under a microscope (magnification, × 100).

The invasion assay followed a procedure similar to that of the migration assay except that the chamber inserts were previously coated with 0.4 mg/ml Matrigel (BD Labware).

### Wound-healing assay

In serum-free medium or medium with 10% FBS, cells were added to six-well plates and cultured for 24 h. Uniform scratches were formed with a 100-µl pipette tip, and cell migration was observed under a light microscope (magnification, × 40) at 0, 24 and 48 h.

### Chromatin immunoprecipitation (ChIP)

The ChIP assay was performed using a ChIP assay kit (Focus Bioscience, Shanghai). The cells were cross-linked by incubation with 1% formaldehyde solution at room temperature, and the reaction was quenched with 140 mM glycine. Subsequently, the chromatin was fragmented into 200–500-bp pieces by sonication. The protein-DNA complexes were incubated with 5 μg of anti-p53 antibody (DO-7; cat. no. SC-47698, Santa Cruz Biotechnology) or negative control IgG (Beyotime, China) and then precipitated with protein G agarose beads. The bound DNA‑protein complexes were eluted. After a series of washes, the cross‑links were reversed. The purified DNA fragments were analysed by real-time PCR. SYBR green master mix (Thermo Scientific, USA) was used for PCR. The sequences of the primers specific for the TRIM11 promoter were as follows: forward, 5′‑TGTCCAAGGGGACCAAGTACTTAT‑3′ and reverse, 5′‑GGACCCATCGCTTACACTGTAG‑3′.

### Statistical analysis

The data from independent experiments are presented as the mean ± SD. Student’s t -test (two-tailed) was used to analyse differences between two groups, and one-way ANOVA was performed with SPSS version 13.0 (SPSS Inc., Chicago, USA) to analyse differences among multiple groups. All the data were collected from three independent experiments. P < 0.05 was considered significant (*).

## Results

### Overexpression of TRIM11 facilitates cell migration and invasion

**Fig. 1:**
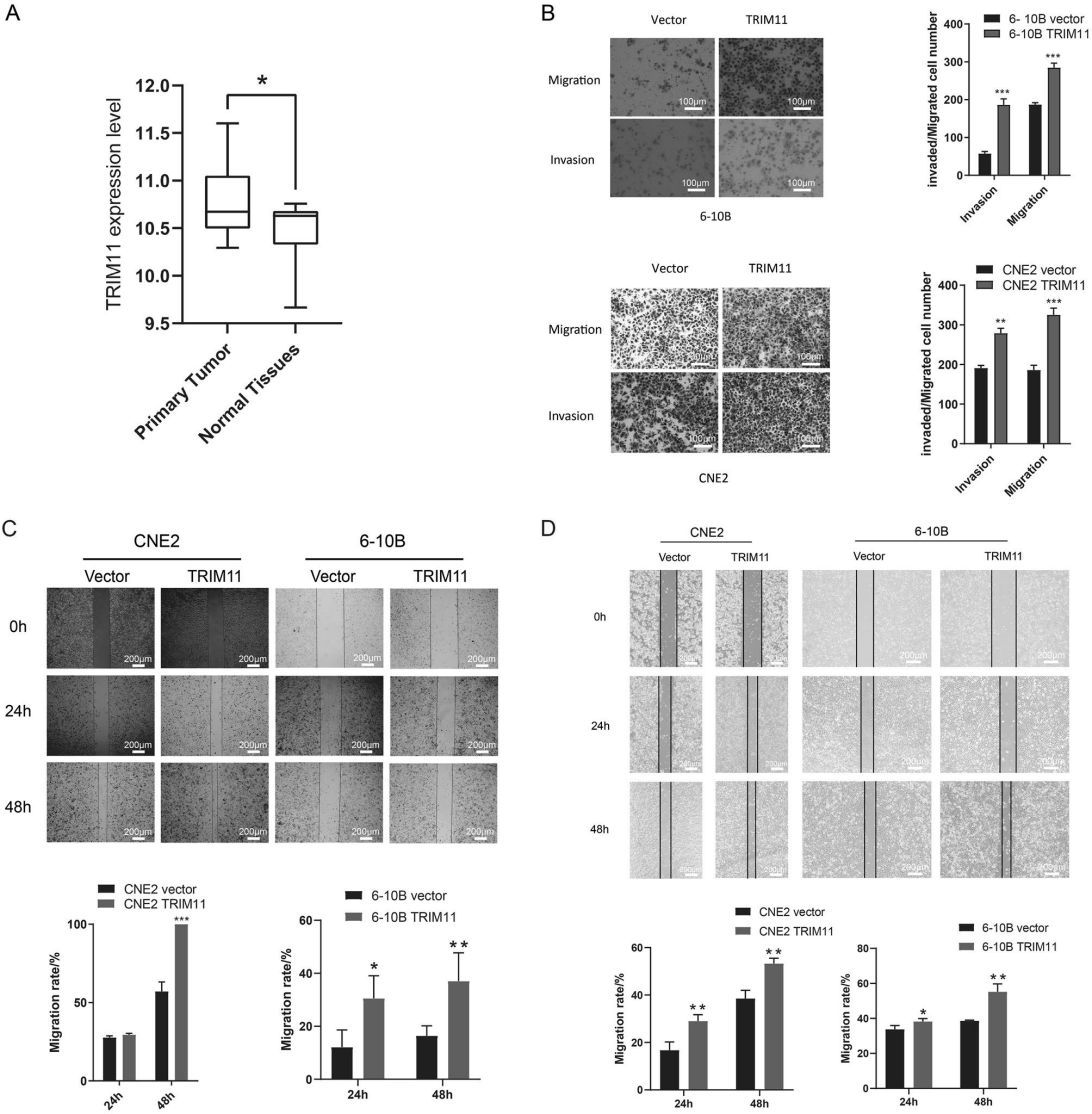
Overexpression of TRIM11 facilitates cell migration and invasion. **A** The expression of TRIM11 in primary tumor and normal tissues. **B** Transwell experiments showing the effects of TRIM11 upregulation on cell migration and invasion. **C** The wound healing ability of CNE2 and 6-10B cells was assessed by wound healing assays in the medium with 10% FBS after 24 and 48 h. **D** The wound healing ability of CNE2 and 6–10B cells was assessed by wound healing assays in serum-free medium after 24 and 48 h. Three individual experiments were carried out. *P < 0.05, **P < 0.01 and ***P < 0.001

To confirm TRIM11 upregulation in NPC, we analysed its expression status using GEO Datasets (GSE53819). We found that TRIM11 was upregulated in NPC tissues (Fig. 1A). Subsequently, our study evaluated the biological function of TRIM11 in NPC cells. Stable overexpression of TRIM11 was established in CNE2 and 6-10B cells. Upregulation of TRIM11 expression significantly enhanced the invasion ability of NPC cells in vitro (Fig. 1B). Furthermore, the migration rate of cells overexpressing TRIM11 was higher, as assessed by wound healing experiments (Fig. 1C,D). In summary, these data suggest that overexpression of TRIM11 promotes NPC cell migration and invasion.

### Downregulation of TRIM11 expression represses the migration and invasion in NPC cells

**Fig. 2:**
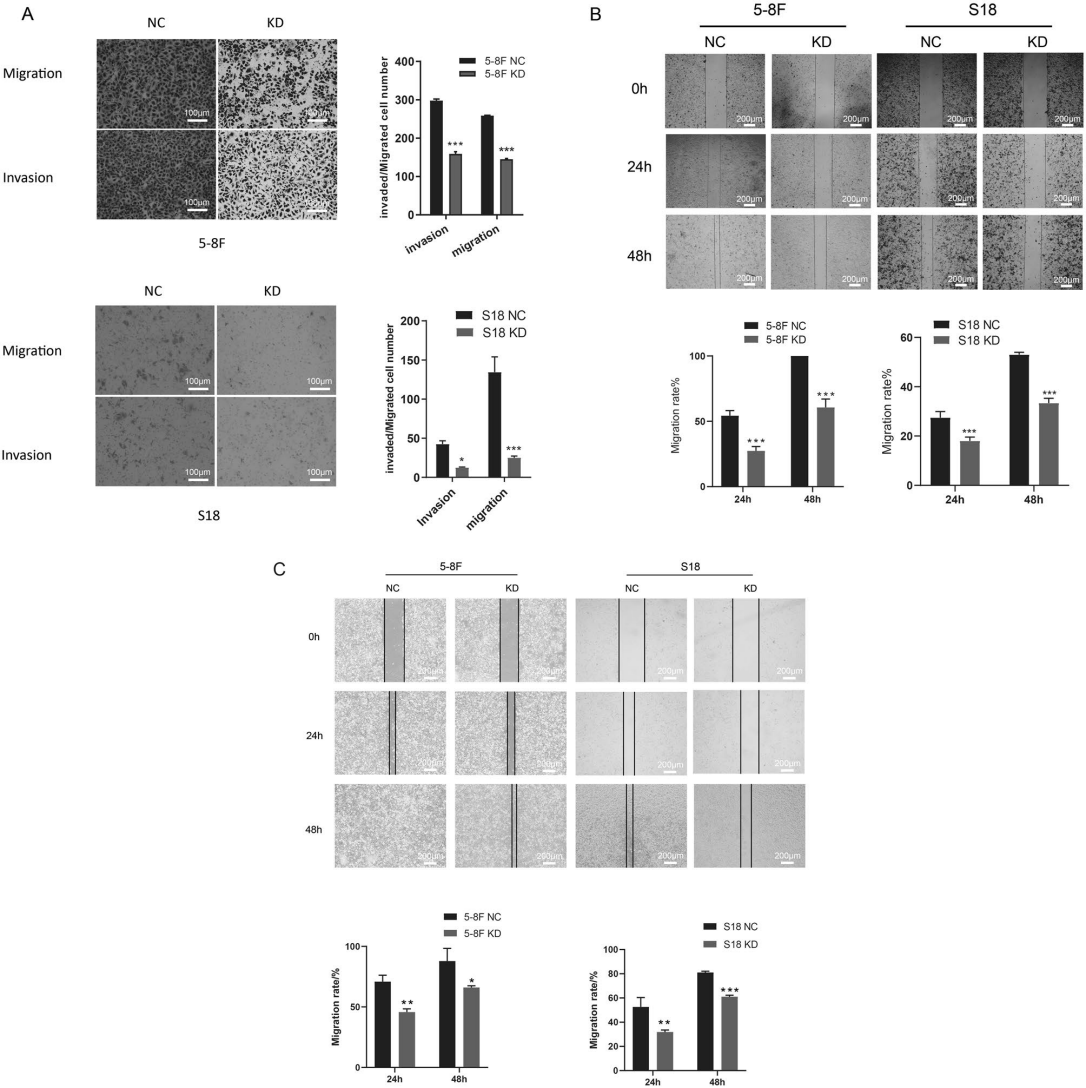
Downregulation of TRIM11 expression represses the migration and invasion in NPC cells. **A** Cell migration and invasion after the downregulation of TRIM11 expression. **B** The wound healing abilities of 5-8F and S18 cells were determined by a wound-healing assay in the medium with 10% FBS after 24 and 48 h. **C** The wound healing abilities of 5-8F and S18 cells were determined by a wound-healing assay in serum-free medium after 24 and 48 h. Three individual experiments were carried out. *P < 0.05, **P < 0.01 and ***P < 0.001

This study further investigated whether cell migration and invasion were significantly reduced by the downregulation of TRIM11 expression in NPC cells. CRISPR-Cas9 gene-editing technology has attracted great interest in cancer research since it promotes the exploration of molecular mechanisms underlying cancer progression and the development of accurate treatment [14]. CRISPR/Cas9 gene editing was used to construct stable TRIM11-knockdown cell lines. We observed that the downregulation group had fewer invasive cells than the control group (Fig. 2A). By assessing the rate of wound healing, we showed that the migration rate of the downregulated group was lower than that of the control group (Fig. 2B, C). These data indicated that decreasing the expression of TRIM11 decreased the migration and invasion abilities of NPC cells.

### p53 inhibits TRIM11 expression by binding to its promoter

**Fig. 3:**
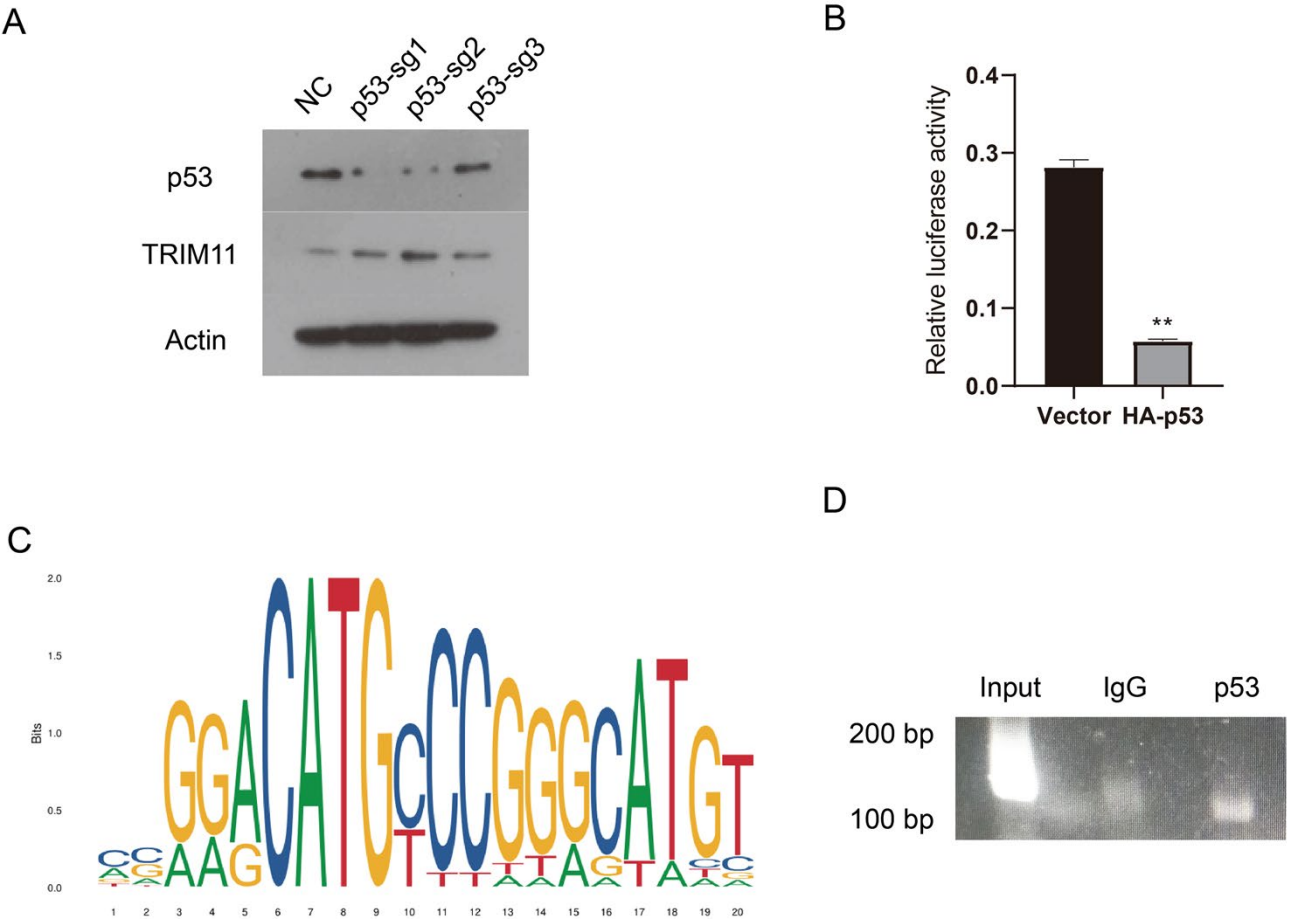
p53 inhibits TRIM11 expression by binding to its promoter. **A** Western blotting confirmed the KD of p53 in CNE2 cells. The TRIM11 protein levels were measured. Actin served as the control. **B** A luciferase reporter assay was carried out to detect luciferase activities in 293T cells. Three individual experiments were carried out. **P < 0.01. **C** The p53 binding site is shown. **D** According to the description in the “Materials and methods” section, cells were analysed by ChIP using an anti-p53 antibody

According to GEO Datasets (GSE53819), the mRNA expression level of TRIM11 is higher in NPC tumor tissues compared to normal tissues. To explore the reason why TRIM11 mRNA expression is upregulated in NPC tumor tissues, we analyze the promoter of TRIM11, which suggested that there was a potential p53 binding site on the TRIM11 promoter. Therefore, we hypothesised that p53 regulates the expression of TRIM11. First, we identified a potential binding site for p53 on the TRIM11 promoter (Fig. 3C). Then, we investigated whether p53 affects the expression of TRIM11 in vitro. p53 was stably knocked down in CNE2 cells using the CRISPR/Cas9 gene-editing technique. As shown in Fig. 3A, KD of p53 increased the expression of TRIM11. Next, luciferase assays were used to reveal the effect of p53 on TRIM11 promoter activity to accurately determine the molecular mechanism by which p53 inhibits TRIM11 expression in NPC. As shown in Fig. 3B, the expression of p53 downregulated TRIM11 promoter activity in 293T cells. Subsequently, we confirmed that p53 directly binds to the promoter of TRIM11 by ChIP (Fig. 3D). It is reported that TP53 is not mutated in CNE2, SUNE1 and 6-10B cells [15]. 5-8F cells are subclones of SUNE1. The cell line S18 is one of 29 clones from the parental cell line CNE2. So, wild type p53 was pulled down in ChIP. The above results show that p53 may inhibit TRIM11 expression by binding to its promoter.

## Discussion

In 2018, an estimated 129,079 cases of NPC were confirmed worldwide, and an estimated 72,987 NPC-related deaths occurred [16]. NPC is a head and neck squamous cell carcinoma with high risks of metastasis and recurrence. Patients with recurrence or metastasis often have an inferior prognosis due to distant metastasis [17, 18]. Multimodality therapy, such as chemotherapy and radiotherapy, has demonstrated efficacy in metastatic and recurrent NPC. On the other hand, immune checkpoint blockage therapies have now emerged as a new therapeutic approach for advanced NPC [19, 20]. Preventing distant metastasis is key to NPC treatment, and more effective systemic drugs should be investigated. This work mainly found that TRIM11 can promote the migration and invasion of NPC cells.

TRIM11 exerts certain carcinogenic effects in a variety of cancers. Di et al*.* found that in primary malignant glioma cultures and high-grade glioma sources, TRIM11 expression was upregulated, and overexpression of TRIM11 may lead to a more aggressive glioma phenotype, increased malignant tumour growth and low survival [21]. On the other hand, Song et al*.* found that TRIM11 can regulate glycolytic metabolism in breast cancer cells [22]. Zhang et al*.* demonstrated that TRIM11 promotes NPC cells resistance to chemotherapy by modulating the β-catenin/ABCC9 axis [23]. However, the phenomenon by which TRIM11 promotes NPC cell migration and invasion is not reported. Here, we demonstrate that TRIM11 is involved in the regulation of the migration and invasion in NPC cells.

p53 is an important tumour suppressor, and the complexities of the function of p53 in regulating cancer cell behaviour are well established [24]. The p53 gene is mutated in more than half of all human cancer cases and almost all types of human cancers [25]. Jackson et al*.* found that mutant p53 promotes *K-ras*–initiated lung adenocarcinomas in a partially dominant-negative manner [26]. Similarly, through both TAp63-dependent and TAp63-independent mechanisms, mutant p53 can enhance MET signals and promote cell invasion [27]. Mutational alterations of the p53 gene in NPC have a distinct signature, which is different from other human cancers. It is estimated that p53 mutation is not frequent in the genomic landscape of NPC [9, 10, 15]. We showed that TRIM11 promoted NPC cell invasion and migration. Interestingly, we found that p53 inhibits TRIM11 expression by binding to its promoter.

## Conclusion

In summary, our study showed that TRIM11 promotes NPC cell migration and invasion in vitro. Mechanistically, p53 inhibits TRIM11 expression by binding to its promoter. In conclusion, our data indicated that TRIM11 plays an important role in the development of NPC and has a certain therapeutic value.
